# Complete Hydatiform Mole with a Coexisting Living Fetus: A Case Report

**DOI:** 10.3390/healthcare13090970

**Published:** 2025-04-23

**Authors:** Irene Piccolotti, Silvia Zago, Maria Paola Bonasoni, Beatrice Rosignoli, Annachiara Boschi, Francesca Lostritto, Francesco Catania, Tiziana Arcangeli

**Affiliations:** 1 Unit of Obstetrics and Gynecology, Azienda Ospedaliero-Universitaria di Ferrara, 44122 Ferrara, Italy; irenepiccolotti@gmail.com (I.P.); rosignoli.bea@gmail.com (B.R.); 2 Pathology Unit, AUSL della Romagna, St. Maria delle Croci Hospital, 48121 Ravenna, Italy; silvia.zago@auslromagna.it; 3 Pathology Unit, Azienda USL-IRCCS di Reggio Emilia, 42122 Reggio Emilia, Italy; 4 Unit of Obstetrics and Gynecology, AUSL della Romagna, St. Maria delle Croci Hospital, 48121 Ravenna, Italy; annachiara.boschi@auslromagna.it (A.B.); francesca.lostritto2@auslromagna.it (F.L.); francesco.catania@auslromagna.it (F.C.); tiziana.arcangeli@auslromagna.it (T.A.)

**Keywords:** complete hydatiform mole with a coexisting living fetus, twin pregnancy, ultrasound, beta-hCG

## Abstract

**Background:** Gestational trophoblastic diseases comprise the hydatiform moles (HMs), complete or partial, an abnormal development of trophoblastic tissue. HMs derive from a gametogenesis error during conception leading to an anomalous chromosomal asset. In the complete hydatiform mole (CHM), when one or two spermatozoa enter an empty oocyte, the karyotype, paternally derived, is diploid 46,XX or 46,XY. CHM is characterized by massive hydropic degeneration of the villi, with no fetal structures, easily detected by ultrasound (US) in early gestation, confirmed by elevated maternal beta-hCG levels. CHM with coexistent fetus (CHMCF) is an exceptional event with a high risk of malignant progression, and severe complications such as massive vaginal bleeding, preeclampsia, and fetal death. **Methods/Results:** We present a case of CHMCF in a 29-year-old woman, which resulted in a liveborn and healthy baby at 38 weeks of gestation. The patient was prenatally carefully monitored with biweekly US and periodic beta-hCG levels. Post-partum follow-up consisted of transvaginal US and beta-hCG levels at 1, 3, and 6 months. After 1 year post-delivery, both the mother and the newborn were healthy. **Conclusions:** CHMCF management can be challenging as shared guidelines are currently lacking and the case described may be helpful in adding more data.

## 1. Introduction

Gestational trophoblastic diseases include a spectrum of rare pre-neoplastic and neoplastic conditions originating from the trophoblastic tissue. Hydatiform moles (HMs), either partial or complete, fall under this definition as benign placental tumours with malignant potential [1,2]. The estimated incidence of HMs ranges from 66 to 121 cases per 100,000 pregnancies in Europe and North America, while it is reported to be higher in Asia, Latin America, and Africa [3].

The main risk factors are extreme maternal age (<15 years and >35 years) and history of a previous molar pregnancy [4].

HM often presents with nonspecific symptoms such as nausea or vaginal bleeding. However, clinical manifestations can be more severe such as heavy vaginal bleeding or watery vaginal discharge due to the passage of hydropic villi, hyperemesis, excessive uterine enlargement for the gestational age, enlarged ovaries due to the presence of theca-lutein cysts, early-onset gestational hypertension, and abnormally high levels of beta-hCG [2,4]. All types of molar pregnancies carry a high risk of maternal and fetal complications: antepartum bleeding, preterm labor, severe preeclampsia, thyrotoxicosis, and intrauterine fetal death [5]. Each type of mole may potentially progress to gestational trophoblastic neoplasia (GTN), which includes a malignant invasive mole, choriocarcinoma, placental site trophoblastic tumor, and epithelioid trophoblastic tumor. In particular, malignant transformation may occur in 15–20% of cases of complete hydatiform mole (CHF), and to a lesser extent in cases of partial hydatiform mole (PHF), estimated at around 0.5–5% [4].

HMs represent an anomaly during gametogenesis or fertilization; they are genetically determined, due to the overexpression of placental genes derived from the paternal genome [6,7]. Based on the number of chromosomes, two different types of HMs have been described: a complete (CHM) and a partial form (PHF).

CHMs usually present with a 46,XX karyotype, all paternal DNA. This chromosomal asset derives from the reduplication of the haploid genome of the sperm and segregation of the maternal chromosomal set. In 5–10% of complete moles, dispermic fertilization may occur, leading to a 46,XY karyotype. Biparental complete moles have been described, but are exceptionally rare [7]. Fetal and vascular structures and red blood cells are typically not detected as the fetus is reabsorbed before the circulatory system develops [2]. Grossly, the CHM is a voluminous specimen with abnormal vesicular villi described as a “bunch of grapes”. Histologically, the villi are diffusely hydropic with circumferential trophoblastic hyperplasia. The implantation site usually displays atypia and exaggerated proliferation [7].

PHMs exhibit a trisomic karyotype resulted from two paternal and one maternal haploid genome. The asset 69,XXX or 69,XXY derives from a haploid ovum fertilized with a replicated single sperm or a dispermic insemination. The 69,XYY karyotype is exceptional and 69,XYY has never been described. Fetal structures, either macroscopic or microscopic, even fetal red blood cells, are always found [2]. In case of an intact triploid fetus, severe symmetrical growth restriction and malformations (cleft palate, spina bifida, renal hypoplasia, syndactyly, and a single transverse palmar crease) are typical [7,8].

Grossly, the placental specimen presents with less evident hydropic villi. Histologically, the villi are of two types: one enlarged with irregular outlines and hydropic changes and one with normal or fibrotic villi. Circumferential trophoblastic hyperplasia may be focal, mild, or moderate [7].

The coexistence of a normal live fetus with a molar gestation, in a twin pregnancy, is almost an anecdotal event, occurring in 1 of 22,000–100,000 pregnancies [2,4,9,10,11,12,13].

This condition represents a health threatening for the mother as it exacerbates the risks of multiple gestations due to the presence of molar tissue.

In the literature, both CHMs and PHMs have been described in association with a twin pregnancy [14], but the majority of cases are in the complete form [15,16]. Cases of partial hydatidiform mole with coexistent fetus (PHMCF) seem to have a lower maternal and fetal risk, including the progression to GTN, than those of complete hydatidiform mole with coexistent fetus (CHMCF) [17].

The majority of these “twin” molar pregnancies are diagnosed antepartum through ultrasound (US), although data from the literature are limited, mainly consisting of single case reports or collective reviews. US typically detects the presence of a cystic placental mass distinct from the fetoplacental unit. More rarely, the diagnosis is made only after delivery by histological examination. According to a study involving patients from Brazil and United States, the percentage of pregnancies complicated by HMs that had resulted in live birth was approximately 60% [12]. However, the risk of GTN progression is reported to be 46%, much higher in patients with elevated beta-hCG levels or symptoms requiring early termination of pregnancy (TOP) [2,12]. Twin pregnancies with HM and a coexisting fetus, compared to singleton molar pregnancies, present an increased risk of developing post-molar GTN and metastases that require multiple chemotherapy treatments [10,11]. Due to the rarity of this condition, the limited data in the literature, and the absence of international guidelines, a twin pregnancy with HM and a living fetus currently remains a challenging issue for obstetricians.

Herein, we present a case of CHMCF, born alive and well. This rare clinical entity deserves particular attention from the obstetrical point of view in terms of maternal–fetal management, and our report may help provide useful guidance for healthcare staff.

## 2. Case Presentation

A 29-year-old Caucasian woman, gravida 2 para 1, was referred to a third level fetal maternal unit at 13 + 0 weeks as during the first-trimester genetic US an abnormal vacuolated area in the gestational sac was noted. This area measured approximately 50 × 60 mm, and it was adjacent to the right placental margin. The fetus was morphologically normal and regularly grown for gestational age. The first-trimester screening test—based on the US measurement of nuchal translucency and maternal serum levels of β-hCG and PAPP-A—indicated a low risk for the three investigated trisomies: trisomy 13, 18, and 21.

The patient had a previous pregnancy with a spontaneous full-term delivery of a healthy infant 8 years earlier. Her medical history was positive only for non-autoimmune hypothyroidism for which she was on replacement therapy (levothyroxine 50 mcg/day). At 6 weeks of pregnancy, TSH was 4.34 mU/L (normal range: 0.200–5.000 mU/L; in pregnancy-related hypothyroidism, the maximum tolerated TSH value is 3 mU/L), and FT4 was 10.5 ng/L (normal range: 8–17 ng/L).

The body mass index (BMI) was 20. The partner’s medical history was unremarkable.

The patient experienced vaginal bleeding a few days before the referral, so she went to the obstetric emergency unit and underwent a beta-hCG test with a result of 415,322 U/L. She was prescribed vaginal progesterone at a dose of 200 mcg per day for two weeks. This indication was then renewed with each subsequent episode of vaginal bleeding.

However, at 13 weeks, she was asymptomatic except for nausea and light vaginal bleeding. Her home therapy included, in addition to treatment with progesterone and levothyroxine, a pregnancy-specific multivitamin containing the recommended amount of folic acid (400 mcg) as well as useful vitamins and minerals (such as B-group vitamins, vitamin C, iron, and magnesium).

**Figure 1:**
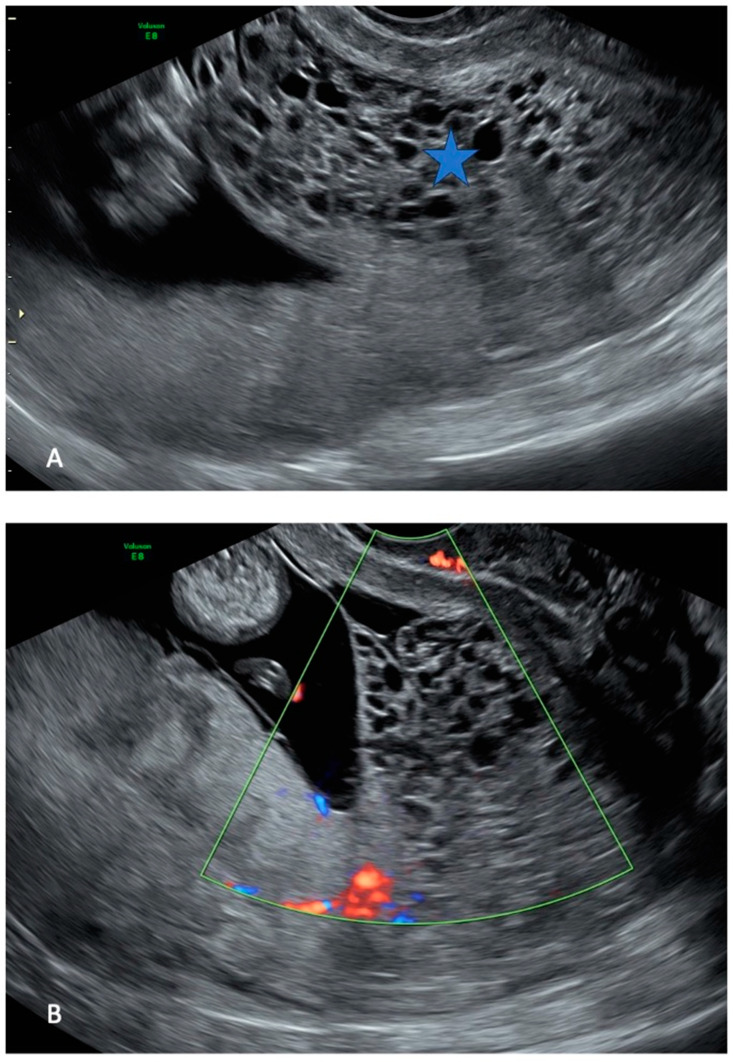
Ultrasound images of hydatiform mole at 13 weeks of gestation: a normal intrauterine pregnancy ((**A**,**B**) left side) coexisted with a multicystic area ((**A**) star, (**B**) echographic cone), compatible with molar degeneration.

The referred US revealed an intrauterine pregnancy with a live fetus of normal size and morphology. US examination showed one placenta with a regular appearance, and adjacent to it, a cystic placental mass, measuring 70 × 50 mm (Figure 1).

Considering the US finding of a second cystic placental mass in the absence of fetal structures, the clinical picture of vaginal bleeding, and the biochemical profile showing elevated maternal beta-hCG levels, a presumptive diagnosis of CHMCF was made. Although the definitive histological diagnosis, which could only have been made through bioptic sampling, was not pursued, the US images were sufficient in providing thorough counseling to the couple regarding maternal risks associated with pregnancy progression or legal TOP. In particular, the couple was informed that, according to data from the literature, the risk of preeclampsia and IUGR associated with the specific clinical condition could have been as high as 25% [9,11].

The couple opted for a conservative approach. Amniocentesis was offered, but declined by the couple. US follow-up was scheduled every two weeks, along with blood tests, including beta-hCG levels, and blood pressure monitoring. A full abdominal US was also planned, the result of which was negative for extra-uterine disease.

At 15 + 0 weeks of gestation, the patient showed a further beta-hCG increase (509,901 U/L) and signs of thyroid hyperstimulation (TSH decreased from 4.34 mU/L in the first weeks of gestation to 0.66 mU/L). For this reason, the endocrinology consultation recommended the discontinuation of levothyroxine.

At 17 weeks of gestation, the molar mass was increased in size, measuring 93 × 65 × 81 mm and laying on the internal cervical os (placenta previa). No evidence of uterine wall invasion was noted.

At 20 weeks of gestational age, the normal pregnancy was unremarkable with a viable and normally grown fetus with no anatomical anomalies, with a placenta located away from the cervix. The molar vesicular area remained stable in size and the patient was asymptomatic except for occasional spotting.

During the following weeks, maternal thyroid function returned to normal limits and levothyroxine therapy (50 mcg/die) was resumed at 22 weeks (TSH 3.79 mU/L). At the same gestational age, the patient presented with intrahepatic cholestasis with a slight elevation in bile acids (13.5 micromol/L with normal values below 10 micromol/L); therefore, ursodeoxycholic acid was started at 300 mg twice a day. The levels of bilirubin, ALT, AST, and alkaline phosphatase were within the normal range.

In the following weeks, the US findings remained unchanged, but at 29 + 2 weeks of gestation, the molar area was no longer previa with the same dimensions.

At 30 weeks, TSH was 3.48 mU/L despite treatment with levothyroxine 50 mcg/day; bile acids were 17.20 micromol/L and alkaline phosphatase was 133 U/L (normal values 35–104 U/L). Bilirubin, ALT, and AST remained within normal limits. Therefore, levothyroxine therapy (75 mcg/die) and ursodeoxycholic acid dosage (300 mg, 3 times/day) were progressively increased. The normal pregnancy proceeded without complications such as preeclampsia or growth restriction.

Starting from 35 weeks, fetal monitoring was carried out with cardiotocography, which was always normal. Elective cesarean section was chosen as mode of delivery due to the risk of retained molar tissue and the patient’s desire.

**Figure 2:**
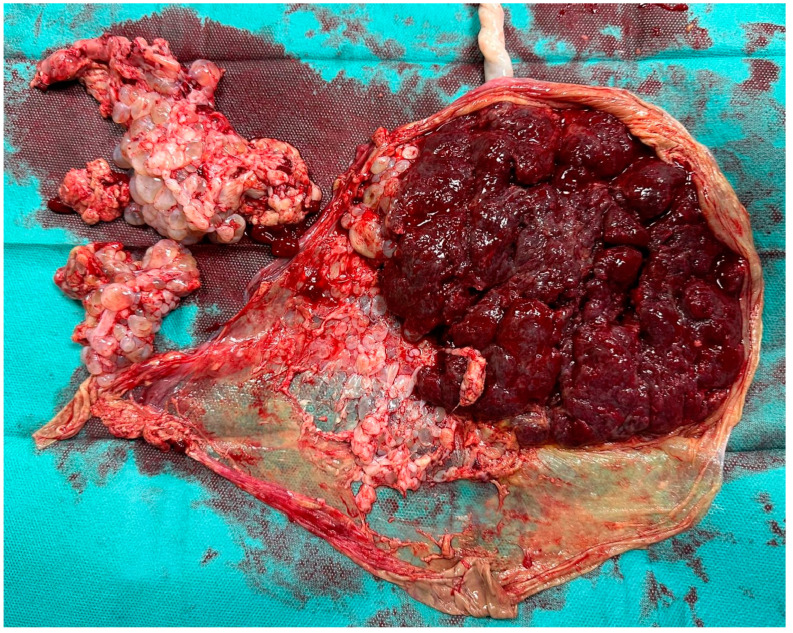
Twin pregnancy with a regular placenta and a coexisting molar degeneration: fresh specimen in the delivery room soon after the cesarean section. The “grape-like” features of the hydropic degeneration of the villi were grossly evident.

At 38 weeks, the patient presented with contractions and was admitted to the obstetric ward. She underwent a cesarean section, resulting in a liveborn male neonate weighing 2660 g, with Apgar scores of 10/10. After surgery, the specimen showed a placenta with a regular appearance and in continuity, a “grape-like” tissue of 70 × 50 mm, consistent with complete molar degeneration (see Figure 2).

Surgery and postpartum were uneventful. During hospitalization, the patient underwent a chest X-ray and an abdominal US, all negative. She was discharged on the third postoperative day.

Follow-up was scheduled with transvaginal US and beta-hCG levels at 1, 3, and 6 months postpartum, all of which were found to be regular with a progressive decrease in beta-hCG levels (see Table 1). After one year from delivery, the mother and the baby were both in healthy conditions.

**Figure 3:**
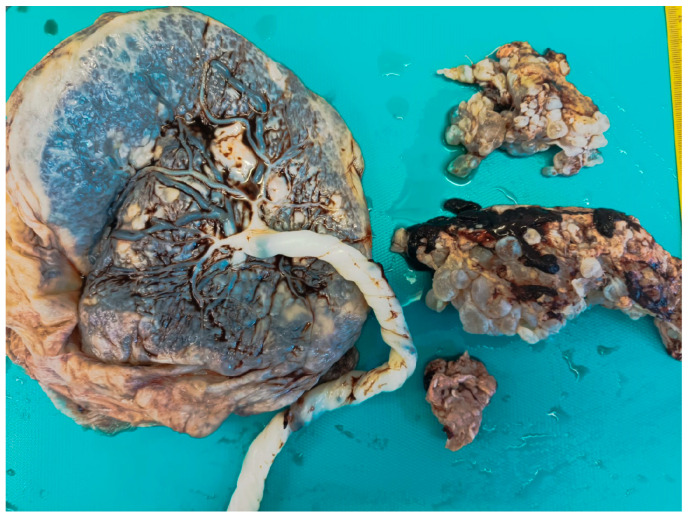
Formalin fixed specimen: a single chorionic disc with a hypercoiled cord coexisted with hydropic villi, macroscopically seen as “grape-like” vesicles.

The placenta was sent for histological examination. It consisted of a single chorionic disc weighing 414 g and measuring 13 × 13 × 1.5 cm. The three-vessel cord was hypercoiled and measured 23 cm in length (Figure 3). The membranes were shredded and the “grape-like” structures were partially attached to the chorionic disc and free in the pot, weighing all together 153 g. At slicing, the placenta showed a focal hematoma, occupying less than 5% of the whole volume.

**Figure 4:**
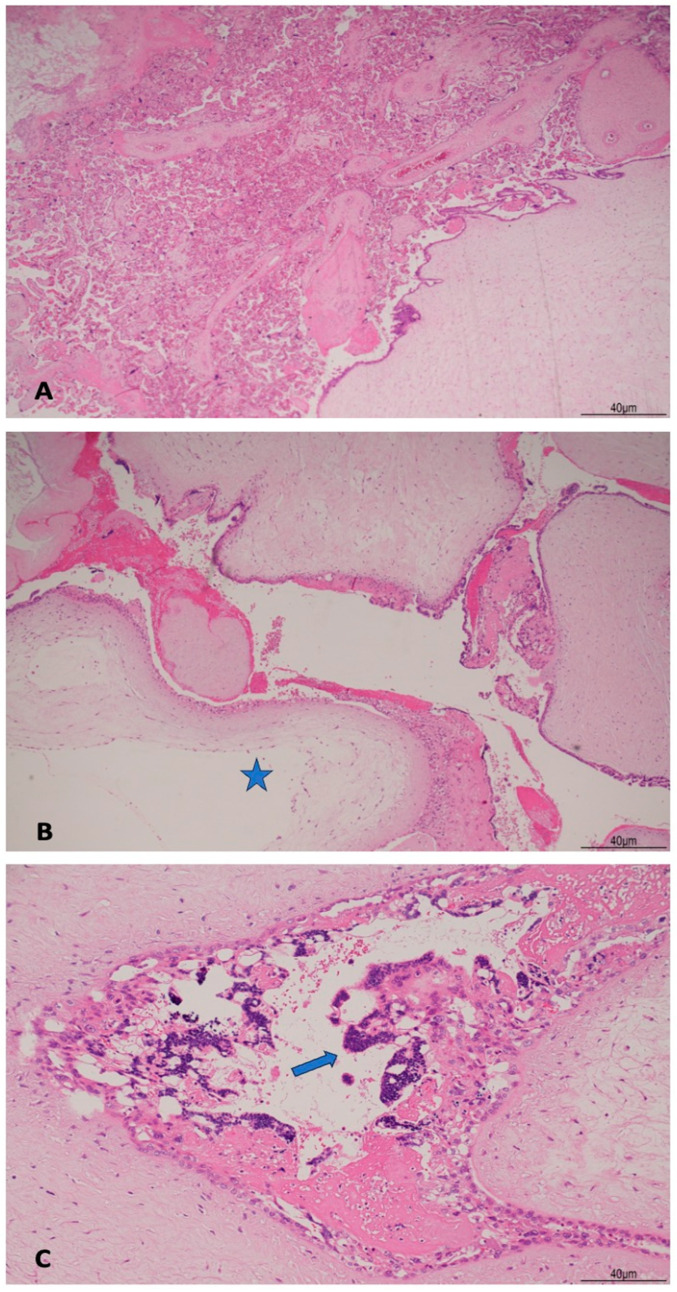
(**A**): Histological findings of coexisting normal pregnancy with hydatiform complete mole: normal placental villi (left side of the image) concurred with villous hydropic degeneration (right bottom side of the picture). There was a sharp passage between normal villi and molar degeneration (hematoxylin and eosin 2HPF). (**B**): The molar component showed enlarged villi with formation of cisterns (star) (hematoxylin and eosin 4HPF). (**C**): The trophoblast was circumferentially hyperplastic with atypia (arrow) (hematoxylin and eosin 10HPF).

**Figure 5:**
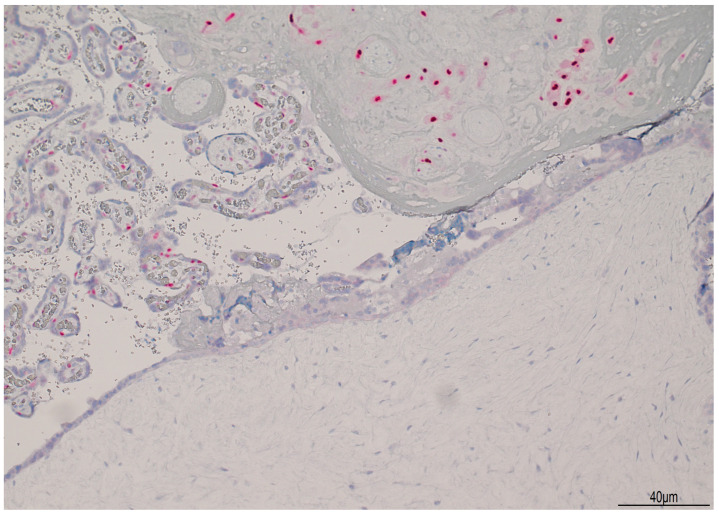
Immunohistochemistry for p57KIP2: in the molar component, p57KIP2 was negative (lower side of the image), but it was positive in the cytotrophoblast of the normal villi and in the intermediate trophoblast (upper part of the picture).

Histologically, the placenta showed a regular villous maturation for 38 weeks of gestational age. The hydropic component showed enlarged and vacuolated villi with well-formed cisterns. The villous trophoblast was circumferentially hyperplastic with atypia involving both cytotrophoblasts and syncytiotrophoblasts (Figure 4). Immunohistochemistry for p57KIP2 was negative in the hydropic component (Figure 5), indicating a CHMCF.

## 3. Discussion

HM occurs with a varying incidence worldwide, ranging from 1–2/1000 pregnancies in North America and Europe to 10/1000 in India and Indonesia [18]. CHMCF is almost exceptional with an incidence of 1 in 20,000 to 100,000 pregnancies [18]. Management of CHMCF can be challenging as maternal complications may occur, especially preeclampsia, hyperthyroidism, and GTN. Intrauterine fetal death and preterm birth are also likely. However, the possibility of GTN is alike between the patients who continue the pregnancy and those who interrupt in the first trimester [5]. A recent meta-analysis [5] investigated the outcome of CHMCF and the rate of liveborn babies increased across the years, reaching a peak of 50% in 2017. The type of delivery, spontaneous or cesarean section, did not affect the onset of GTN. In case of CHMCF, the most common maternal complications appear to be vaginal bleeding and early preeclampsia. As a matter of fact, no correlation has been found between beta-hCG levels and maternal symptoms, and/or progression to GTN [19].

Currently, there are no shared guidelines for the management of patients with CHMCF who decide to continue their pregnancy. Good clinical practice should recommend careful and frequent check-ups and surveillance by a multidisciplinary team composed of a gynecologist, a neonatologist, and an oncologist [5].

However, since CHMCF represents a rare occurrence, management and parental counselling are still a challenge.

In the revised literature, the outcome of CHMCF has been mainly reported as aggregated data, summarized in Table 2. Sebire et al. [11] investigated 77 cases of CHMCF, of which 20 resulted in livebirths (25.9%) and the others were terminated or resulted in spontaneous abortion. GTD was detected in 31 pregnancies (40.2%), 19 of which were interrupted and 12 of which were in women who continued. In their study, although CHMCF showed a high risk of miscarriage, the risk of GTD was not increased between the women who decided to terminate their pregnancy and those who opted to carry on.

Wee et al. [9], in their review, reported only a 25% of chance of livebirth and a 35% risk of developing GTD. However, in ongoing pregnancies, early onset preeclampsia (20%) and fetal loss (29%) were observed.

In a wide study by Lin et al. [12], 72 CHMCF pregnancies were analyzed, and 35 (60%) resulted in a healthy newborn. The other pregnancies (37) resulted in early termination, spontaneous abortion, or IUFD. The overall incidence of GTD was 46% (31 cases), statistically associated with higher levels of beta-hCG and medical complications.

Massardier et al. [20] reported a series of 14 cases with CHMCF, of which only 3 (21%) ended up with a healthy newborn. One was born premature at 25 weeks and died after 3 days due to sepsis. GTD was recorded in 7 patients (50%).

In a case series by Giorgione et al. [21], 6/13 patients delivered a viable baby, but one of them died soon after birth. A total of 4/13 women (31%) experienced GTD.

The FIGO Cancer report 2018 highlighted the high percentage of livebirths (40–60%) in CHMCF, although the risk of GTD is around 20% [22].

In a paper by Hemida et al. [19], 12 molar pregnancies with a coexisting live fetus resulted in three live babies (25%); one of them died after 5 months due to multiple congenital anomalies. In all pregnancies, even in the liveborns, the authors did not differentiate between partial and complete moles, since histology was available only in 10/12 of cases and they were all complete.

The broadest study was conducted by Hajri et al. [23], in which 141 pregnancies with CHMCF were thoroughly investigated. Pregnancy termination or spontaneous miscarriage before the 24th week was recorded in 90 cases. Livebirth was reached in 51/141 (36%) and 43/51 newborns survived after 8 days. GTD was recorded in 35/136 women (26%) as 5 patients were lost during follow-up.

**Table 2:** Summary of studies on CHMCF reporting aggregated data.

Reference	Number of CHMCF	TOP, Miscarriage, IUFD	Livebirths	Neonatal Outcome	GTN
Sebire et al. [11]	77	57	20	--	31 (19 interrupted and 12 continued)
Lin et al. [12]	72	37	35	--	31
Massardier et al. [20]	14	11	3	1 premature baby at 25 weeks, deceased after 3 days for sepsis	7
Giorgione et al. [21]	13	6	6	1 early neonatal death	5
Hajri et al. [23]	141	90	51	8 deaths within 8 days	35/136 women; 5 patients not available in the follow-up period
Piura et al. [17]	31	1	28	2 neonatal deaths	5

CHMCF: complete hydatiform mole with coexistent fetus; TOP: termination of pregnancy; GTN: gestational trophoblastic neoplasia.

Single case reports of CHMCF are more discordant as they include early TOP or abortion and live babies. Piura et al. [17], reviewed the previous literature, documenting 31 cases at term, including theirs, of which 28 cases involved live babies, excluding 1 stillborn and 2 neonatal deaths. GTD was documented in 5 women. Unfortunately, information was not fully available in all cases, but, interestingly, in most women beta-hCG levels reached a peak in the second trimester, then decreased. The authors concluded that in cases of CHMCF expectant management could be an acceptable possibility instead of TOP, but close fetal and maternal surveillance must be mandatory.

In the case we described, a case of CHMCF, the patient was managed by obstetricians, resulting in the birth at term of a healthy neonate via cesarean section without major maternal complications. During the course of the pregnancy, the patient experienced a phase of hyperthyroidism in the first and early second trimesters, which later resolved, returning to her pre-gestational hypothyroid state. She also developed mild intrahepatic cholestasis of pregnancy and urticarial eczema, which resolved before delivery. The patient remained normotensive throughout the pregnancy and experienced only gestational nausea in the first and second trimesters and vaginal spotting. The primary challenge for the healthcare staff was establishing the correct timing for prenatal monitoring in the absence of clear guidelines. The clinical approach included biweekly US, comprehensive blood tests with thyroid and hepatobiliary function assessments, and measurement of beta-hCG levels. These investigations successfully monitored pregnancy progression, allowing the early detection of clinical and laboratory changes leading to appropriate treatment. Postpartum follow-up consisted of transvaginal ultrasound and beta-hCG monitoring at 1, 3, and 6 months after delivery. Chest X-rays, and abdominal and pelvic US were also scheduled. This approach was fundamental in the potential early detection of any US or laboratory signs of disease persistence or recurrence. Regarding the patient’s future reproductive life, before attempting another pregnancy, it was recommended waiting at least 6 months after beta-hCG normalization. In case of pregnancy, early transvaginal US was also advised due to the increased risk of molar recurrence.

Differential diagnosis between PHMCF and CHMCF is important as the latter carries a higher risk of maternal complications and progression to GTN. Post-molar GTN may occur in 15–20% cases of CHM, reducing to 1.5% in case of PHM [24]. US is the gold standard in detecting molar degeneration and the diagnosis can be made around the 12–14th week. In CHM, the placenta presents numerous cysts (the “snowstorm” or “clusters of grapes”) and the gestational sac is enlarged with no embryo. In PHM, the increased placental echogenicity is more scattered due to focal hydropic degeneration [25]. Beta-hCG levels are necessary to make the diagnosis of mole, but they do not discriminate between CHF and PHM. Thus, the correct diagnosis is made postnatally integrating US and pathological examination [21]. Morphology and p57KIP2 immunohistochemistry are still time- and cost-effective tools in differential diagnosis. In CHM, nuclear positivity of p57KIP2 is present only in the intermediate trophoblast and in the decidua, but its expression is absent in the villous cytotrophoblast. Instead, in PHM, p57KIP2 is strongly positive in the cytotrophoblast nuclei, other than the other sites aforementioned [7]. Immunohistochemical expression of p57 can be explained as the *p57KIP2* gene, a cyclin-dependent kinase inhibitor, located on chromosome 11p15.5, is subject to strong paternal imprinting and is expressed from the maternal allele. As CHM is mostly paternally derived and lacks a maternal genome, p57KIP2 is absent in the villous cytotrophoblast and in stromal cells. On the other hand, in PHM, as it contains maternal DNA, p57KIP2 is instead positive. In both type of moles, hydropic abortion, and in normal pregnancy, p57KIP2 is always positive in the decidua and extra-villous trophoblast. Syncytiotrophoblast cells are always negative [26]. Molecular genotyping can be helpful in differentiating between androgenetic and biparental complete mole, and also in identifying germline mutations in *NLRP7* and *KHDC3L* genes, responsible for recurrent mole [2].

In the case we described, US detection of CHMCF was made early in gestation, at 13 weeks. Placental examination confirmed the US diagnosis, both grossly and microscopically. The enlarged, hydropic villi were adjacent to a normal placenta. Histologically, the typical cisterns with circumferential trophoblast hyperplasia were seen and immunohistochemistry for p57KIP2 was negative in the cytotrophoblast and stromal cells.

## 4. Conclusions

The significant challenge of this case was the clinical management of the patient, as shared guidelines are currently lacking and data from the literature are limited. However, prenatal and postnatal accurate and close-up monitoring resulted in an excellent maternal and fetal outcome.

On the whole, this case and its management add further valuable information to the rarity of CHMCF, in order to better identify its natural history and prognosis.

## Figures and Tables

**Table 1:** Maternal beta-hCG levels during pregnancy and after delivery.

Pregnancy	
**Gestational age (weeks + days)**	**Value (U/L)**
12 + 4	369,926.0
12 + 6	415,322.0
14 + 2	509,915.0
16 + 3	486,825.0
**Puerperium**	
**Days after delivery**	**Value (U/L)**
22	57.4
28	64.0
35	60.3
41	42.2
49	30.2
56	16.1
63	13.4
71	7.0
83	3.5
90	2.1
106	0.8
129	0.3
161	<0.1

## Data Availability

The data presented in this study are available on request from the corresponding author.

## References

[1] Chawla T., Bouchard-Fortier G., Turashvili G., Osborne R., Hack K., Glanc P. (2023). Gestational Trophoblastic Disease: An Update. Abdom. Radiol..

[2] Soper J.T. (2021). Gestational Trophoblastic Disease: Current Evaluation and Management. Obstet. Gynecol..

[3] Altieri A., Franceschi S., Ferlay J., Smith J., La Vecchia C. (2003). Epidemiology and Aetiology of Gestational Trophoblastic Diseases. Lancet Oncol..

[4] Ngan H.Y.S., Seckl M.J., Berkowitz R.S., Xiang Y., Golfier F., Sekharan P.K., Lurain J.R., Massuger L. (2018). Update on the Diagnosis and Management of Gestational Trophoblastic Disease. Int. J. Gynaecol. Obstet. Off. Organ Int. Fed. Gynaecol. Obstet..

[5] Wang G., Cao J., Xu X., Han X., Cui H. (2022). Delivery Management of a Complete Hydatidiform Mole and Co-Existing Viable Fetus: A Meta-Analysis and Systematic Review. J. Gynecol. Obstet. Hum. Reprod..

[6] Fisher R.A., Khatoon R., Paradinas F.J., Roberts A.P., Newlands E.S. (2000). Repetitive Complete Hydatidiform Mole Can be Biparental in Origin and Either Male or Female. Hum. Reprod..

[7] Kaur B. (2021). Pathology of Gestational Trophoblastic Disease (GTD). Best Pract. Res. Clin. Obstet. Gynaecol..

[8] Daniel A., Wu Z., Bennetts B., Slater H., Osborn R., Jackson J., Pupko V., Nelson J., Watson G., Cooke-Yarborough C. (2001). Karyotype, Phenotype and Parental Origin in 19 Cases of Triploidy. Prenat. Diagn..

[9] Wee L., Jauniaux E. (2005). Prenatal Diagnosis and Management of Twin Pregnancies Complicated by a Co-Existing Molar Pregnancy. Prenat. Diagn..

[10] Fishman D.A., Padilla L.A., Keh P., Cohen L., Frederiksen M., Lurain J.R. (1998). Management of Twin Pregnancies Consisting of a Complete Hydatidiform Mole and Normal Fetus. Obstet. Gynecol..

[11] Sebire N.J., Foskett M., Paradinas F.J., Fisher R.A., Francis R.J., Short D., Newlands E.S., Seckl M.J. (2002). Outcome of Twin Pregnancies with Complete Hydatidiform Mole and Healthy Co-Twin. Lancet.

[12] Lin L.H., Maestá I., Braga A., Sun S.Y., Fushida K., Francisco R.P.V., Elias K.M., Horowitz N., Goldstein D.P., Berkowitz R.S. (2017). Multiple Pregnancies with Complete Mole and Coexisting Normal Fetus in North and South America: A Retrospective Multicenter Cohort and Literature Review. Gynecol. Oncol..

[13] Yayna A.A., Ayza A., Dana W.W., Yemaneh A., Worana B., Tesfaye A., Desalegn A., Kassaye G., Adamu E., Leka Y.A. (2023). Complete Molar Pregnancy with Coexisting Normal Twin Complicated by Hyperthyroidism That Resulted in Normal Alive Delivery: A Case Report from Wolaita Sodo, Ethiopia. SAGE Open Med. Case Rep..

[14] Jacobs P.A., Szulman A.E., Funkhouser J., Matsuura J.S., Wilson C.C. (1982). Human Triploidy: Relationship between Parental Origin of the Additional Haploid Complement and Development of Partial Hydatidiform Mole. Ann. Hum. Genet..

[15] Imafuku H., Miyahara Y., Ebina Y., Yamada H. (2018). Ultrasound and MRI Findings of Twin Pregnancies with Complete Hydatidiform Mole and Coexisting Normal Fetus: Two Case Reports. Kobe J. Med. Sci..

[16] Vaisbuch E., Ben-Arie A., Dgani R., Perlman S., Sokolovsky N., Hagay Z. (2005). Twin Pregnancy Consisting of a Complete Hydatidiform Mole and Co-Existent Fetus: Report of Two Cases and Review of Literature. Gynecol. Oncol..

[17] Piura B., Rabinovich A., Hershkovitz R., Maor E., Mazor M. (2008). Twin Pregnancy with a Complete Hydatidiform Mole and Surviving Co-Existent Fetus. Arch. Gynecol. Obstet..

[18] Joyce C.M., Fitzgerald B., McCarthy T.V., Coulter J., O’Donoghue K. (2022). Advances in the Diagnosis and Early Management of Gestational Trophoblastic Disease. BMJ Med..

[19] Hemida R., Khashaba E., Zalata K. (2022). Molar Pregnancy with a Coexisting Living Fetus: A Case Series. BMC Pregnancy Childbirth.

[20] Massardier J., Golfier F., Journet D., Frappart L., Zalaquett M., Schott A.M., Lenoir V.T., Dupuis O., Hajri T., Raudrant D. (2009). Twin Pregnancy with Complete Hydatidiform Mole and Coexistent Fetus: Obstetrical and Oncological Outcomes in a Series of 14 Cases. Eur. J. Obstet. Gynecol. Reprod. Biol..

[21] Giorgione V., Cavoretto P., Cormio G., Valsecchi L., Vimercati A., De Gennaro A., Rabaiotti E., Candiani M., Mangili G. (2016). Prenatal Diagnosis of Twin Pregnancies with Complete Hydatidiform Mole and Coexistent Normal Fetus: A Series of 13 Cases. Gynecol. Obstet. Investig..

[22] Melamed N., Baschat A., Yinon Y., Athanasiadis A., Mecacci F., Figueras F., Berghella V., Nazareth A., Tahlak M., McIntyre H.D. (2021). FIGO (International Federation of Gynecology and Obstetrics) Initiative on Fetal Growth: Best Practice Advice for Screening, Diagnosis, and Management of Fetal Growth Restriction. Int. J. Gynaecol. Obstet. Off. Organ Int. Fed. Gynaecol. Obstet..

[23] Hajri T., Massoud M., Vergne M., Descargues P., Allias F., You B., Lotz J.-P., Haesebaert J., Bolze P.-A., Golfier F. (2024). Multiple Pregnancy with Complete Hydatidiform Mole and Coexisting Normal Fetus in a Retrospective Cohort of 141 Patients. Am. J. Obstet. Gynecol..

[24] Braga A., Andrade T., do Carmo Borges de Souza M., Campos V., Freitas F., Maestá I., Sun S.Y., Pedrotti L.G., Bessel M., Junior J.A. (2023). Presentation, Medical Complications and Development of Gestational Trophoblastic Neoplasia of Hydatidiform Mole after Intracytoplasmic Sperm Injection as Compared to Hydatidiform Mole after Spontaneous Conception—A Retrospective Cohort Study and Literature Review. Gynecol. Oncol..

[25] Cavoretto P., Cioffi R., Mangili G., Petrone M., Bergamini A., Rabaiotti E., Valsecchi L., Candiani M., Seckl M.J. (2020). A Pictorial Ultrasound Essay of Gestational Trophoblastic Disease. J. Ultrasound Med..

[26] Sarmadi S., Izadi-Mood N., Abbasi A., Sanii S. (2011). p57KIP2 Immunohistochemical Expression: A Useful Diagnostic Tool in Discrimination between Complete Hydatidiform Mole and Its Mimics. Arch. Gynecol. Obstet..

